# Hybrid Approaches for Selective Laser Sintering by Building on Dissimilar Materials

**DOI:** 10.3390/ma13225285

**Published:** 2020-11-22

**Authors:** Babette Goetzendorfer, Thomas Mohr, Ralf Hellmann

**Affiliations:** Applied Laser and Photonics Group, University of Applied Sciences Aschaffenburg, Wuerzburger Str. 45, 63743 Aschaffenburg, Germany; mohr.thomas93@gmail.com (T.M.); ralf.hellmann@th-ab.de (R.H.)

**Keywords:** hybrid additive manufacturing, selective laser sintering, multicomponent process

## Abstract

We introduced a new approach in selective laser sintering for hybrid multicomponent systems by fabricating the sintered polyamide 12 (PA12) part directly onto a similar (PA12) or dissimilar (polyamide 6 (PA6) and tool steel 1.2709) joining partner. Thus, the need for adhesive substances or joining pressure was completely circumvented, leading to the possibility of pure hybrid lightweight bi-polymer or metal–polymer systems. By taking advantage of the heating capabilities of the sinter laser regarding the substrate surface, different exposure strategies circumvented the lack of overlapping melting temperatures of dissimilar polymers. Therefore, even sintering on non-PA12 polymers was made possible. Finally, the transfer on metallic substrates—made up by selective laser melting (SLM)—was successfully performed, closing the gap between two powder-based additive processes, selective laser sintering (SLS) and SLM.

## 1. Introduction

Hybrid components consisting of metal and polymer have recently gained considerable attention in numerous sectors of industry such as automotive [1,2,3], electronics [4], medical [5], or aerospace engineering [6,7]. By combining the advantages of both materials, entirely new functional characteristics are attainable [8,9]. In accordance with the intended application, for example, lightweight components, different aspects such as weight, improved mechanical behavior, adapted thermal conductivity, and lower production cost might be considered [8,10].

Several approaches have been made for developing multicomponent parts, most of which assemble two finished elements by joining them, e.g., through mechanical fastening or the use of an adhesive [11,12,13]. Unfortunately, mechanically joined hybrid components using processes such as riveting, screwing, or jacking often exhibit disadvantageous load transmission, which may destabilize the joining area [14,15]. Adhesive technologies may circumvent this drawback. However, introducing adhesives is a sensitive issue, for example, in biomedical applications, and increases production complexity and costs.

Laser-based joining has been put forth as an alternative to adhesives [16,17]. However, as this joining process requires specific joining conditions such as an initial surface treatment [18] and the application of pressure [19], complex structures for, e.g., sophisticated lightweight applications cannot be processed by using this technique.

A possible approach to realizing hybrid, adhesive-free, lightweight components is additive manufacturing (AM) for at least one of the joining partners [20]. Employing the advantages of AM [21,22,23,24], several attempts at hybrid fabrications have been made and reviewed for multi-material applications by Vaezi et al. [25]. Additionally, a combination of additive metal manufacturing and conventional substractive processes such as milling has been demonstrated [26,27]. Moreover, two different additive technologies have been combined in hybrid approaches, such as stereolithography with selective laser melting (SLM) [28,29], one-photon and two-photon polymerization [30], and multijet combined with Fused Deposition Modelling (FDM) [29]. Furthermore, FDM on a metallic surface has been demonstrated to obtain hybrid objects [31]. In general, hybrid structures consisting of metal and polymer elements reveal a significant dependence of the joining quality on the surface structure of the metallic mating part as shown in [25,27,29,32,33,34], as the connection area is characterized by mechanical binding effects.

Against this background, one aim of this study is to investigate the suitability of SLM-built metal devices as part of hybrid polymer–metal objects, as this technology offers the possibility to produce controlled surface geometries of various types [35,36]. Pretreatment processes to modify conventional metallic surfaces can thus be circumvented, facilitating experimental investigations of surface binding correlations.

As a polymer partner, (SLS) as an industrially well-established additive manufacturing technology was introduced as an AM process to produce hybrid structures. First, SLS of standard polyamide (PA12) on similar material was performed. Then, the process was adapted in order to combine different polyamides (PA12 on PA6). After that, the application was extended to dissimilar material partners by sintering PA12 on metal parts that, in turn, had been built by selective laser melting.

## 2. Materials and Methods

**Figure 1:**
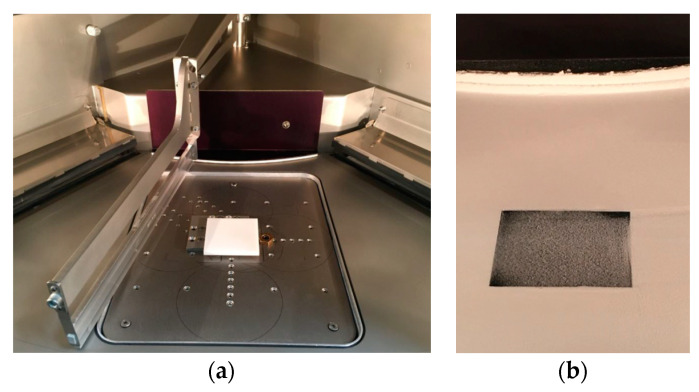
(**a**): Polyamide 12 (PA12) substrate fixed onto the custom-built ground plate. (**b**): PA6 substrate embedded in PA12.

SLS was performed using an EOS Formiga P110 (EOS GmbH, Krailling, Germany) sintering PA12 polyamide standard material (PA2200, EOS GmbH, Krailling, Germany) with a 30 W CO_2_ laser (spot diameter, 500 µm). A custom-built ground plate allowed for the implementation of different basic substrates (PA12, PA6 (smapla GmbH, Koblenz, Germany), SLM part) into the building chamber (Figure 1). First, the substrate was mounted onto the custom-built ground plate (Figure 1a) to generate a defined and fixed basic area. Second, the remaining space was filled with PA12 powder to level out the total build plate for the first building layer. Figure 1b shows a mounted PA6 substrate (black) embedded in PA12 powder, prepared for starting the hybrid process at layer height zero.

**Figure 2:**
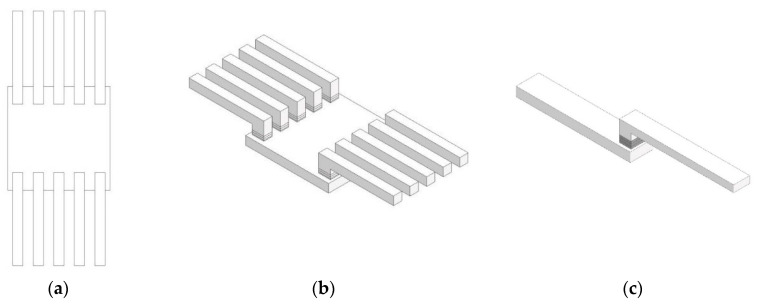
Geometry of sintered test specimen on basic substrate plate. (**a**) Top view. (**b**) Perspective view. After sintering, the hybrid print plate is separated into ten single test specimens. (**c**) Reference sample as benchmark object (binding layers 5 × 7 mm^2^ grey).

To access and evaluate the joining strength of sintered material on different base plates, tensile shear tests in end lap adhesive sealing geometry were performed according to DIN EN 1465 [37], as illustrated in Figure 2a,b. The DIN standard is mainly used for comparative purposes and therefore gave the opportunity to judge and evaluate the different hybrid processes within this study. Nevertheless, the described hybrid approach was not defined in a standard procedure until now, so that adaption of adhesive standards was necessary.

Tensile shear tests were performed using a universal testing machine (Xplus, Shimadzu AG, Shimadzu Deutschland GmbH, Duisburg, Germany). A test method analogue to tensile shear tests for laser-joined specimen (5 mm/min) was chosen due to the very similar geometric structure. The resulting maximum force was normalized to the joining area of 5 × 7 mm^2^.

For comparison reasons, reference PA12 test structures were developed and characterized. Figure 2c depicts the geometry of these specimens, which were printed with standard building parameters en bloc in contrast to the aimed hybrid process (basic plates thickness: 5 mm; joining area: 5 × 7 mm^2^). A tensile shear strength of 25.9 MPa (σ = 0.58) was obtained, which can be defined as maximum value for the given geometry, as the continuous building process should lead to optimal mechanical properties. It is worthwhile to stress that all tested hybrid-built specimens broke outside the latter contact area of the hybrid objects (highlighted grey layers in Figure 2c), proving that the specific test geometry of the specimen does not represent a predetermined breaking point itself.

As the shear strength of hybrid components is directly linked with the quality of the joining area, the building parameters within the very first layers are determinative, and thus, the energy density of these layers was experimentally varied. For SLS build parts, the mechanical, e.g., tensile, properties are sensitive to and depend on the deposited energy absorbed by the powder during laser scanning [38,39]. The amount of deposited energy E during the CO_2_-laser process can be calculated by the applied laser average power P divided by the product of scanning speed v and hatch distance H [38]. In this study, we kept the hatch distance constant at 0.2 mm, since on the one hand larger distances lead to porous textures with inferior mechanical properties, and on the other hand smaller distances result in deformation of the built structure. Several studies have analyzed the dependence of the melting temperature on the energy density in the SLS process [40,41,42,43,44], providing typical energy densities in the range of 16–50 mJ/mm^2^ for stable sintering processes. While varying the scanning speed up to 3500 mm/s and adapting the laser power to maintain the applied energy density leads to comparable results in terms of part quality and mechanical properties, we chose 1500 mm/s as an intermediate scanning speed that facilitates sufficiently good results by varying only the laser power and thereby covers the preferable energy density.

In our study, the mechanical strength of the hybrid parts was determined by the quality of the contact zone between substrate and molten-layered PA12 powder, analogue to single material SLS parts described in [45]. With a PA12 layer height of 100 µm and the penetration depth of the CO_2_-laser (emission wavelength 10.6 μm) in the order of 120 µm for standard PA12 [46], the notable binding area comprised the surface of the basic plate and the first three sintered layers at most. Starting from layer 4, the sintering parameters did not affect the binding area any further, so that parameter variation was not required and standard building parameters (EOS values are unpublished) lead to constant properties within the test specimen.

As the layer thickness of the very first layer could be reduced to 50 µm, the impact of the laser on the very first contact layer could be additionally increased. This lower limit of 50 µm was given by the machine accuracy and the PA12 powder distribution [47]. The particle size distribution (PSD) of the used PA12 powder was investigated with a Retsch Camsizer X2, based on dynamic digital image analysis methods according to ISO 13322-2. The found diameters D10, D50, and D90 corresponded, respectively, to the particle sizes equal or lower to which the 10%, 50%, and 90% of the PSD was enclosed. Here, D10 (36.7 µm), D50 (51.7 µm), and D90 (69.4 µm) were in well agreement with previous PA2200 powder characterizations [48], underlining that enough particles smaller than 50 µm were available to create a 50 µm layer.

**Figure 3:**
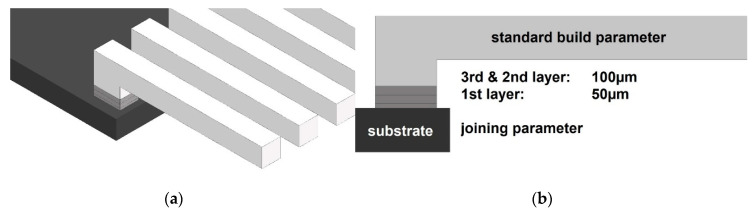
Schematic view. ((**a**) Perspective view, (**b**) side view) on the three binding layers (dark grey).

In Figure 3, the schematic view on the connection layers is illustrated. Variation of the first binding layer height from 50, 80, 100, and 120 µm resulted in reproducible binding effects for 50 and 80 µm. Thicker layers in the range of the penetration depth prevented reliable binding due to insufficient energy density on the contact area. Therefore, a first layer height of 50 µm was chosen for the following experimental study. To additionally evaluate the influence of the applied fluence, the tensile shear strength was determined for energy densities in the range between 17 and 83 mJ/mm^2^.

As basic substrates, PA12 plates were sintered on the used EOS Formiga P110 with standard parameters, whereas PA6 substrates were injection-molded plates. To implement a metallic part, SLM plates (steel tool 1.2709) were produced on a DMG Mori LaserTec 30 (DMG Mori AG, Bielefeld, Germany). By adjusting the energy density on a slightly deficient energy level, leading to insufficient layer melting, a stochastically porous surface structure of the SLM part was obtained. Optical measurements were performed using a Keyence VR-3200 profilometer (Keyence Deutschland GmbH, Neu-Isenburg, Germany).

## 3. Results and Discussion

**Figure 4:**
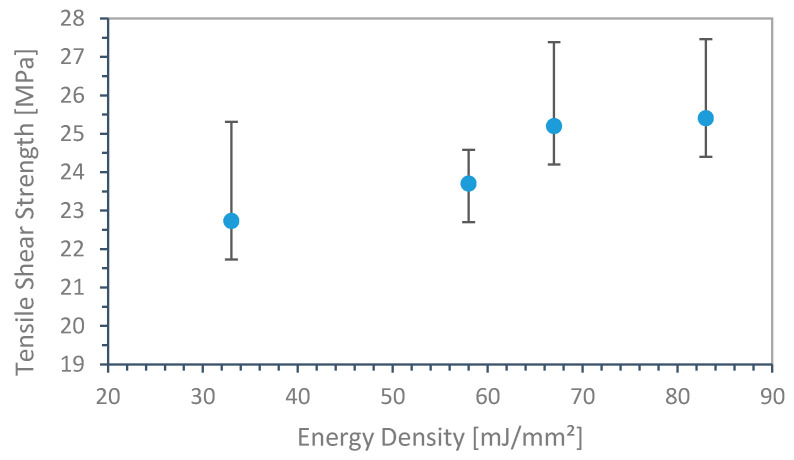
Tensile shear strength of hybrid produced similar material specimen (PA12 on PA12).

In a first attempt, SLS was performed on similar base material (PA12). A seperately sintered PA12 basic substrate was mounted into the build chamber (Figure 1), and the PA12 join partner was sintered onto the surface. Figure 4 depicts the tensile shear strength as a function of applied energy density. Hatch distance (0.2 mm) and scan speed (1500 mm/s) were kept constant. Below 33 mJ/mm^2^, no binding occurs.

In the studied range, the resulting tensile shear strength revealed only a minor dependence, varying from 23 to 25 MPa, on the applied energy density, indicating no distinct correlation. As the internal reference specimen exhibited maximum shear strength of 25.9 MPa, the obtained values reached near optimal binding behavior.

In order to investigate the effect of the scan speed, ten different speed values ranging from 250 to 3500 mm/s with constant energy density of 33 mJ/mm^2^ were considered. Within this range, the resulting tensile shear strength was 24.7 MPa (σ = 1.52).

**Figure 5:**
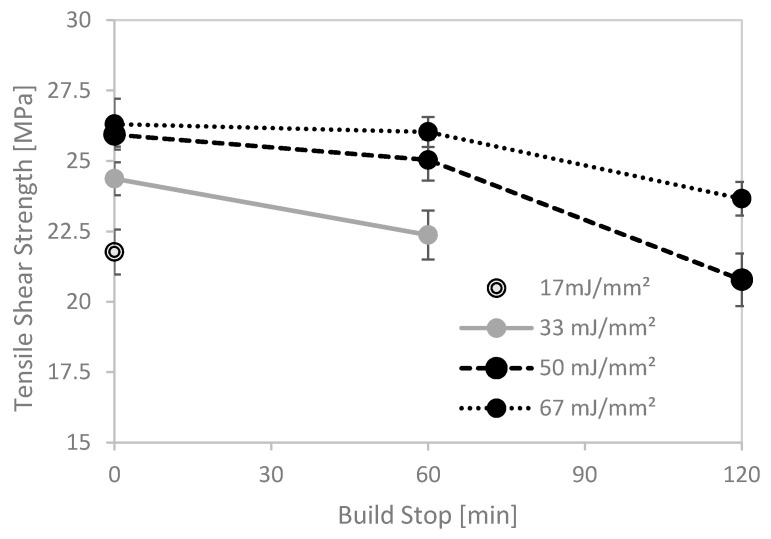
Tensile shear strength of reference samples, imitating the hybrid building process.

As the original hybrid approach included two components (basic plate and sintered part) made via the same SLS process, a reference experiment was performed. First, the basic plate structure was sintered; then, the process was paused for 0/60/120 min, respectively. Followed by that, the three crucial binding layers (Figure 3, grey) were sintered with defined building parameters. Starting with layer 4, the standard building parameters were used as described above. Figure 5 depicts the resulting shear strength values.

With no interruption to the build process, all four energy densities lead to a stable object. The tensile shear strength rose with higher energy densities, implying the known correlation between binding layer parameters and mechanical properties in our hybrid system as well. If a timeout of 60 min was forced before sintering of the first binding layer started, an energy density of 17 mJ/mm^2^ was no longer sufficient to build a stable hybrid object. Further, the tensile shear strength at higher energy densities was reduced. For even longer interruptions (120 min), this effect became more pronounced, with no binding at all for fluences below 50 mJ/mm^2^.

Notably, all samples whose build process was interrupted broke at the joining area, despite relatively high tensile shear strength. We, therefore, assume that interrupting the building process leads to a kind of predetermined breaking point. This effect may be partially attributed to a shrinkage of the sintered PA12, having a higher density (0.93 g/cm^3^) as pulverulent PA12 (0.43 g/cm^3^). As a result, the height of the first powder layer increases, which in turn will be less fused, leading to an inferior bond. Thus, these findings support our previous assumption that the formation and thickness of the first layer has a major influence on the resulting mechanical properties of the bond of the hybrid compound.

**Figure 6:**
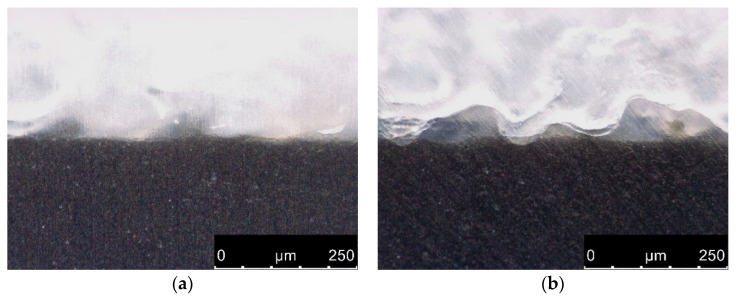
Binding area of PA12 (white) on PA6 (black) in dependence of preceding laser exposure. (**a**) Without creation of a local melt pool a clear boundary line is visible. (**b**) After 6 exposure cycles the dark grey fusion domain is enlarged and the interfacial area becomes blurred, increasing the inter-polymer bonding.

To transfer the approach to dissimilar materials, subsequently, PA12 was sintered onto PA6 substrates. As the melting point of PA6 (210–225 °C [49,50]) is significantly higher than for PA12 (178 °C), sintering laser parameters used above for joining PA12 and PA12 could not be applied, which led to a weak bond with a resulting tensile shear strength of only 3.5 MPa. To vanquish this inherent difficulty, the CO_2_ sintering laser was used as a heating source for the plain PA6 surface to generate a preheated and molten surface without applying a powder layer. This approach compensated for the different melting points of the two polymers, as PA12 powder was applied on a softened surface to enhance the bonding properties of the first powder layer. Figure 6 shows the binding area of PA6 (black) and PA12 (white) with (Figure 6b) and without (Figure 6a) pre-creation of a local melting pool by preceding exposure cycles.

**Figure 7:**
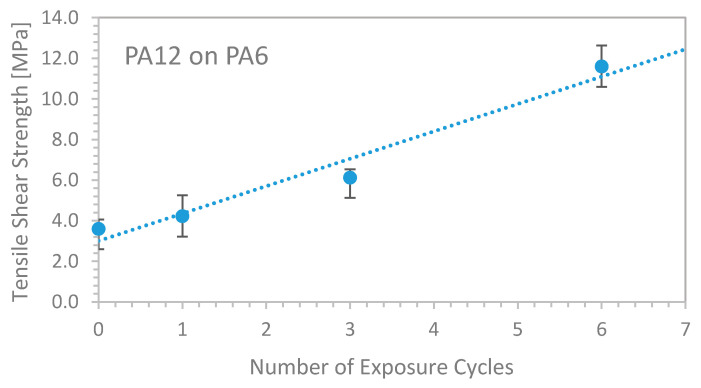
Tensile shear strength of PA12 sintered on PA6 as a function of preceding exposure cycles.

The resulting tensile shear strength increased linearly with the number of preceding exposure cycles as summarized in Figure 7, reaching 11.4 MPa for six exposure cycles prior to applying the first joining powder layer, corresponding to an increase by more than a factor of three. For more than six preceding exposure cycles, the amount of molten PA6 exceeded a critical level, modifying planarity of the surface so much as to hinder a proper and even coating with PA12 powder. The energy density varied from 33 to 67 mJ/mm^2^, but as determined before, the effect of energy fluence variation became superimposed by the dominant impact of the binding layer formation.

**Figure 8:**
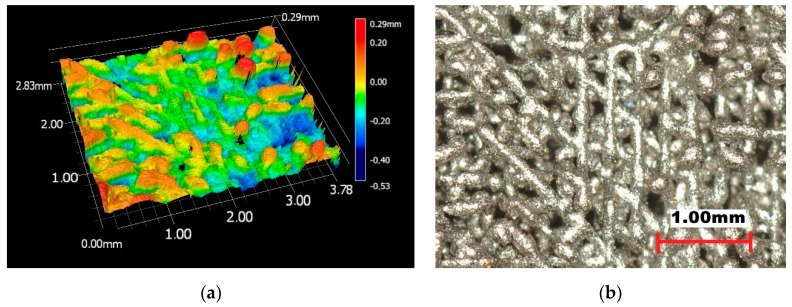
Surface of selective laser melting (SLM)-built metal substrate. (**a**): 3D view. (**b**): Top view.

For the hybrid sintering approach to join dissimilar PA12 onto a selectively molten metal substrate, the sintering strategy had to be adapted, as the melting temperature, apparently, differed significantly. Here, the inherent surface roughness, cavities and channels of the SLM-created metal surface, intentionally created by adjusting the energy density to a deficient energy level, lead to insufficient layer melting, yielding mechanical interlocked bonding between PA12 and the metal surface. In detail, a large hatch distance (0.3 mm compared to 0.115 mm standard hatch) was used in combination with an insufficient melting irradiation by introducing non-illuminated layers within the building process and diminished laser energy. Therefore, the applied energy was only about 25% of the energy which is needed to obtain full density objects. Figure 8a depicts a 3D view of the metallic surface. Figure 8b shows the top view.

**Figure 9:**
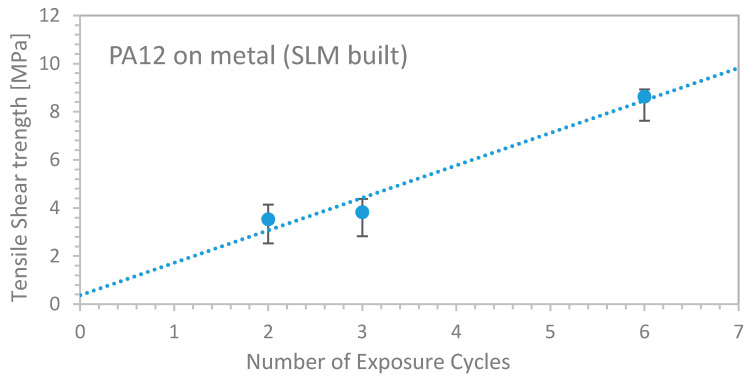
Tensile shear strength of PA12 sintered on SLM steel 1.2709 basic plate as a function of preceding exposure cycles.

The SLM steel 1.2709 substrate was built by DMG Mori LaserTec 30 and manually placed into the SLS machine. The custom-built ground plate (Figure 1a) facilitated proper fixing and correct levelling of the substrate. Subsequently, a PA12 powder layer was manually applied onto the metallic surface before starting the printing process. The polymer powder trickled into the surface cavities and was then molten by the CO_2_ laser irradiation. Upon its subsequent solidification, the polymer created a mechanical bonding to the metallic substrate, hooking into the porous structure. As described before, several exposure cycles were performed to establish a tight interconnection. Figure 9 shows the resulting tensile shear strength as a function of the preceding exposure cycles.

**Figure 10:**
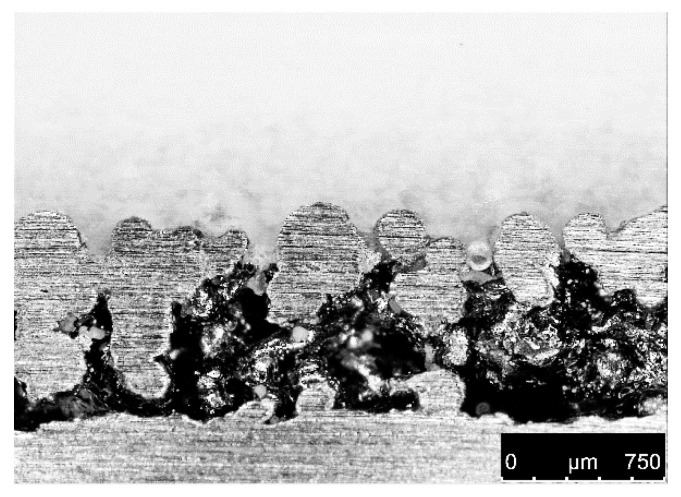
Cross-section of SLM basic plate with molten PA12 (6 exposure cycles).

Apparently, the tensile shear strength increased linearly nearly threefold from 3 MPa (two exposure cycles) up to 8.5 MPa (six exposure cycles). Figure 10 shows a microscope image of a cross-section through the hybrid part, where the thickness of the interlocking area can be determined at about 300 µm. Below that, no molten PA12 structure was produced.

**Figure 11:**
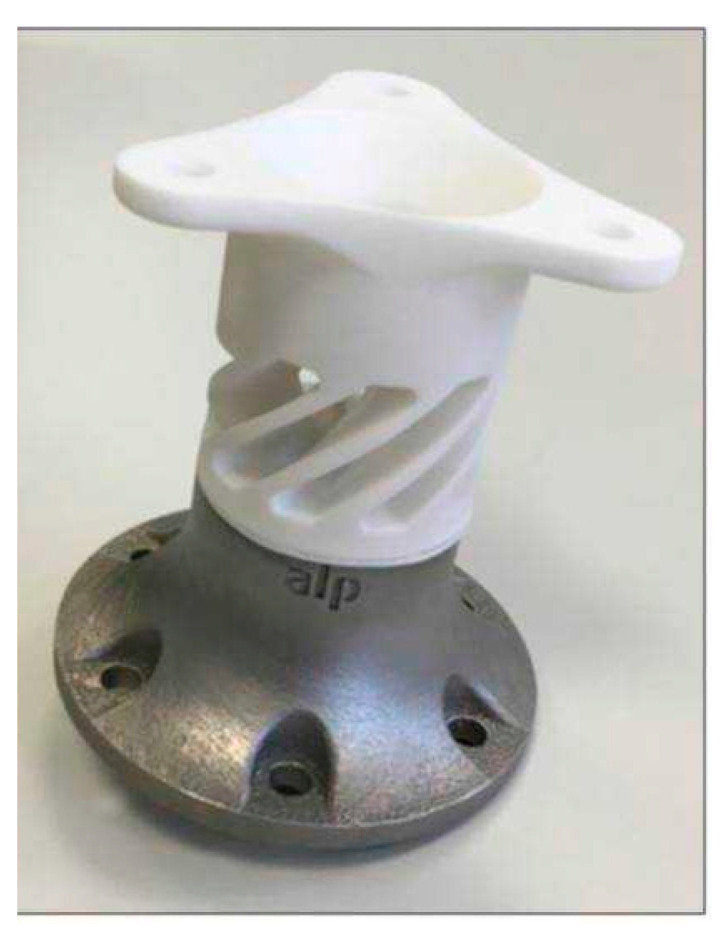
Dynamic overload clutch fabricated by the new polymer–metal hybrid process.

To illustrate the potential of hybrid selective laser sintering of a PA12 lightweight structure onto what may as well be a 3D-printed metal lightweight substrate (selective laser melting), Figure 11 highlights a dynamic overload clutch as a hybrid metal–polymer device. By producing a conventional metallic basic element by SLM, internal lightweight structures reduce the material usage and the overall weight by a significant amount. Moreover, the surface structure can be optimized for hybrid sintering. In addition, the fins of the upper polymer part can be individually adapted to specific load dimensions. The combination of two powder-based additive manufacturing processes uses the lightweight advantages to full capacity, considering specific requirements regarding stability and complex design.

## 4. Conclusions

This contribution introduced a novel approach for hybrid selective laser sintering on different similar and dissimilar substrate materials. In particular, sintering on external build PA12 substrates was demonstrated, with the resulting tensile shear strength being on the order of continuously sintered PA12 reference specimen. In addition, sintering PA12 on dissimilar, non-PA12-polymer substrates with divergent melting temperatures was facilitated by the development of a new strategy in laser exposure. The initial creation of a local melt pool by the CO_2_ sintering laser enhanced bonding between, in our study, PA12 on a PA6-substrate by a factor of three, as quantified by the tensile shear strength. Finally, this approach is extended to the formation of a hybrid metal–polymer object, with the metal substrate itself being additively manufactured by selective laser melting. By melting the first powder layer into the amplified cavities on the metal surface, an interlock system was generated, leading to strong mechanical bonding effects. Appropriate surface structures can easily be obtained by the use of selective laser melting.
